# The Spectrum of Molecular Pathways in Gliomas—An Up-to-Date Review

**DOI:** 10.3390/biomedicines11082281

**Published:** 2023-08-16

**Authors:** Reinhold Nafe, Elke Hattingen

**Affiliations:** Department of Neuroradiology, Clinics of Johann Wolfgang Goethe-University, Schleusenweg 2-16, D-60528 Frankfurt am Main, Germany; elke.hattingen@kgu.de

**Keywords:** signaling pathways, tyrosine kinase receptors, tumor protein 53, hippo pathway, angiogenesis, apoptosis, non-coding RNAs, circular RNAs, micro-RNAs

## Abstract

During the last 20 years, molecular alterations have gained increasing significance in the diagnosis and biological assessment of tumors. Gliomas represent the largest group of tumors of the central nervous system, and the main aim of this review is to present the current knowledge on molecular pathways and their alterations in gliomas. A wide range of new insights has been gained, including evidence for the involvement of the WNT pathway or the hippo pathway in the pathobiology of gliomas, indicating a broad involvement of different pathways formerly not considered to play a central role in gliomas. Even new aspects of angiogenic, apoptotic, and metabolic pathways are presented, as well as the rapidly growing field of epigenetic processes, including non-coding RNAs. The two major conclusions drawn from the present review are the distinct interconnectivity of the whole spectrum of molecular pathways and the prominent role of non-coding RNAs, especially circular RNAs, in the regulation of specific targets. All these new insights are discussed, even considering the topic of the resistance to therapy of gliomas, along with aspects that are still incompletely understood, like the role of hydroxymethylation, or even ferroptosis, in the pathobiology of gliomas.

## 1. Introduction

Gliomas are the most frequent brain tumors, representing approximately 80% of all tumors of the central nervous system [1,2]. In recent years, a growing number of molecular alterations have been described in this tumor group and, therefore, a comprehensive up-to-date overview of the molecular pathways and their major alterations in gliomas is worthwhile. The main purpose of this review is to provide a good assessment of the diversity and interconnectivity of these pathways, as well as an assessment of the individual molecular alterations and their significance in glioma growth. New aspects that are currently of high interest are highlighted, starting with important signaling receptors, which are not limited to the group of receptor tyrosine kinases [3]. Signaling pathways represent a large part of the spectrum of molecular cascades that are altered in gliomas, in addition to the pathways of angiogenesis and apoptosis, and even the metabolic pathways of glucose, glutamine, the citrate cycle, and lipids [1,3,4,5]. Furthermore this study addresses the observation that epigenetic processes play a growing part in our understanding of tumors, since they appear to have a predominant role in influencing the other molecular pathways. In addition to the now well-known importance of DNA methylation and histone modifications in tumor biology, this especially applies to non-coding RNAs (ncRNAs), among which, in particular, the circular RNAs (circRNAs) seem to exert a kind of control function, even on other ncRNAs, such as microRNAs (miRNAs) [6]. 

## 2. Receptors and Different Modes of Their Activation

The overexpression of the tyrosine kinase receptors (RTKs) and their ligands is among the most frequent alterations in glioma tumorigenesis, leading to alternate autocrine or paracrine stimulatory loops and to the aberrant activation of RTKs and their downstream molecular pathways. The RTKs consist of a transmembrane domain, an extracellular ligand-binding domain, an intracellular tyrosine kinase domain, and tyrosine residues leading to downstream activation after their phosphorylation. Due to a variety of molecular changes, such as oncogenic mutations, the silencing of tumor-suppressor genes, gene amplification, or epigenetic changes, overactivated RTKs may contribute to the oncogenic phenotype. The effects of this dysregulation include the overexpression of growth factors or RTKs themselves, or even their involvement in autosomal genetic or epigenetic changes, such as mutations, chromosomal rearrangements, or promotor methylation [2,3,7]. Over 50 different RTKs have been identified in humans; the major receptors overexpressed in many gliomas are the epidermal growth factor receptor (EGFR), the platelet-derived growth factor receptor (PDGFR), the insulin-like growth factor receptor 1 (IGF1R), the fibroblast growth factor receptor (FGFR), and the vascular endothelial-growth factor receptor (VEGFR). Far more RTKs have been demonstrated to be upregulated in gliomas, and their common feature is the ability of each RTK to activate more than a single downstream pathway, so that different activation modes of RTKs lead to the same effect, which is the overactivation of these pathways, promoting tumorigenesis [2,3,8,9]. Additionally, there is increasing evidence that RTKs may not only be activated by their natural ligands, but also through alternative modes, such as calcium signaling or G-protein-coupled receptor signaling. Calcium signaling is a major factor in tumorigenesis in general, and the depletion of Ca^++^ by disrupting its mobilization in vitro or in experimental models provided evidence for reduced tumor cell invasion and for the extended survival of animals with different tumor types, including glioblastomas [3,10,11]. The G-protein-coupled receptors (GPCRs) can lead to the autocrine or paracrine stimulation of RTKs by means of the formation of heteroreceptor complexes or through interactions with the intracellular domain of RTKs. The promotion of glioma cell growth and RTK activation was confirmed in vitro, as well as an additional role of GPCRs in glioma angiogenesis [12,13,14]. The Shc adaptor proteins (“proto-oncogenic Src homology and collagen-proteins Shc”) represent another protein family, from which ShcD can induce ligand-independent phosphorylation and, thus, the activation of tyrosine kinase receptor EGFR. Associated with another tyrosine kinase receptor (Tie2), in vitro studies revealed the synergistic effect of ShcD and Tie2 with the promotion of glioma cell invasion. The blockage of this invasion was demonstrated for glioma cells with the ShcD-mutant protein, which cannot bind and hyperphosphorylate Tie2. The association of ShcD with RTKs is therefore considered a further targetable signaling axis in glioma progression [15,16]. Focal adhesion kinase (FAK) is a protein kinase involved in cellular adhesion and signal-transduction pathways due to its activation by means of autophosphorylation, or even its mediation by integrins. In its autophosphorylated state, FAK binds to Src family kinases (“Cellular-sarcoma family kinases Src”) and activated FAK/Src complexes, and then initiate downstream pathways promoting glioma cell growth, invasion, and even angiogenesis. In vitro and experimental studies using inhibitors of FAK autophosphorylation have demonstrated reduced glioma growth and the reduced invasivity of tumor cells, as well as longer survival of the tumor xenografted *mice* [17,18]. An inverse relationship between FAK and the activity of ShcD and Tie2 was demonstrated in vitro in U87 glioma cells, since ShcD and Tie2 coexpression was associated with the dephosphorylation of focal adhesion kinase FAK. This result suggests the mutual regulation and control of FAK autophosphorylation by ShcD and Tie2 [3,15]. Integrins are transmembrane receptors that are involved in the integration of cellular signals and the surrounding stroma and are thus an important additional type of transmembrane receptor; they are capable of activating tumor-promoting downstream pathways through processes within the extracellular environment. Integrins are known to play an important role in the invasive behavior of malignant gliomas, but the specific role of the different integrins is still under investigation. An important observation is that the expression of the transforming growth factor beta-induced (TGFBI) by tumor-associated macrophages promotes glioma stem cell maintenance and glioblastoma growth via integrin αvβ5-Src-Stat3 signaling [19]. Other integrins, such as α3-integrin, have also been confirmed to be overexpressed in glioma stem cells and to promote glioma invasion in vitro [20]. Based on the observation that glioma stem cells, but not normal astrocytes, are sensitive to lysis by natural killer cells in vitro, an experimental study with the inhibition of αv-integrin and TGF-ß signaling together with allogenic NK treatment showed an improvement in the function of natural killer cells against glioblastoma stem cells, preventing tumor growth in glioma stem-cell-transplanted *mice* [21]. Of note, CD44 is a transmembrane receptor typical of a hyaluronic acid-rich cellular environment and shares the important role of promoting glioma growth and invasion with integrins [22]. Although traditionally considered mediators of immune cell migration, chemokines and their receptors are now also considered to be involved in glioma cell migration and tumor growth. The upregulation of the CXCL8-CXCR1/2 and CXCL12-CXCR4 chemokine axes in glioma cells are important examples. The so-called “atypical chemokine receptors” are also of growing interest, and there is already evidence of their role in glioma growth and even tumor angiogenesis [23,24,25,26].

## 3. Signaling Pathways

Overactivation of receptors by their corresponding ligands or also by alternative or pathological stimuli such as mutations leads to the increased stimulation of various signaling pathways that contribute to the pathogenesis and progression of gliomas. All of these signaling pathways have a complex network of connections, confirming their role in numerous cellular processes. A good example is the PI3K/AKT/PTEN pathway, which is involved in several processes such as proliferation, glucose metabolism, or cell survival. The first step within the activated pathway is the recruitment of phosphatidylinositol 3-kinase (PI3K), which phosphorylates phosphatidyl inositol bisphosphate to triphosphate (PIP2 to PIP3). Normally, PTEN (phosphatase and tensin homologue) blocks PiP3 signaling and AKT (protein kinase B) activation, inhibiting proliferation. Frequent PTEN mutations in glioblastomas lead to a blockade of this inhibition and a complete activation of this signaling pathway with its main effector molecule, the serine/threonine kinase mTOR (mammalian target of rapamycin). mTOR itself leads to a promotion of cell growth and cytoskeletal organization through the activation of the multiprotein complexes, mTORC1 and mTORC2 [3,27,28,29]. The mitogen-activated kinase pathway including the G-protein RAS (“rat sarcoma”) can be activated by the same receptors and alterations as other RTK pathways. This RAS/MAPK signaling pathway also plays a central role in gliomagenesis, particularly in low-grade pediatric-type gliomas. The signaling cascade starts with RAS and extends to major components such as rapidly accelerated fibrosarcoma type B (BRAF), extracellular regulated mitogen-activated protein kinase (MEK), and extracellular signal-regulated kinases (ERK), which promote cell cycle progression and anti-apoptosis genes. Two important aspects underline the central role of this signaling pathway in tumorigenesis: the tumor-suppressor gene *NF1* encodes neurofibromin, which is a physiological suppressor of RAS, and NF1 mutations are responsible for the absence of this natural suppression. *BRAF gene* mutations such as BRAFV600E lead to overactivation of the MAPK pathway, which has led to successful attempts to target this mutation with dabrafenib or trametinib in children and adult patients [2,3,30]. The tumor protein p53 is part of the TP53/MDM2/MDM4 pathway, which becomes active in response to DNA damage or other cellular changes. TP53 then activates proteins such as p21 to inhibit the transition from G1 to the S phase of the cell cycle. Other physiological functions of TP53 contribute to the regulation of apoptosis and cellular senescence. Mouse double minute homologs 2 and 4 (MDM2 and MDM4) are negative regulators of TP53, while MDM2 itself is activated by TP53 and negatively regulated by p14ARF. Therefore, the loss of normal function of TP53 may be due to alterations of different proteins within the signaling pathway. The central effect of this impaired normal TP53 function is the loss of cell cycle control and the TP53-mediated induction of apoptosis [29,31,32,33]. TP53 is a central protein in the process of oncogenesis, and it is emphasized that TP53 protein is likely mediated by a number of interacting effector functions rather than a single pathway or individual transcriptional targets, many of which remain to be uncovered in the future [33]. Like TP53 under physiological conditions, the retinoblastoma protein pRB1 also inhibits the transition from G1 to the S phase to prevent the cell from replicating damaged DNA. The p16^INK4a^/CDK4/RB1 pathway often shows alterations in gliomas. Inactivation of pRB1 through its phosphorylation mediated by CDK4/6 is one way to promote tumor progression. This activation of the cell cycle transition from G1 to the S phase is supported by an increased release of the transcription factor E2F. p16^INK4a^ and even cyclin-dependent kinase 2 types A and B (CDKN2A/B) are regulators of CDK4/6-mediated pRB1 phosphorylation so that the loss of normal function of pRB1 can be triggered by alteration of either of these proteins. The interconnectivity of all these pathways is confirmed by the fact that key molecules such as CDKN2A/B are involved in the p16^INK4a^/CDK4/RB1 pathway and the TP53/MDM2/MDM4 pathway [29,31,34]. The JAK/STAT pathway is also linked to other signaling pathways, as it can transactivate the PI3K/AKT/PTEN pathway and the RAS/MAPK pathway [35]. Its central protein families are the Janus kinases (JAKs) and the signal transducers and activators of transcription proteins (STATs), which are a signal transduction pathway from the cell membrane to the nucleus due to activation by growth factors and cytokines, such as interleukins and interferons. In general, JAKs are activated by the binding of ligands, leading to the phosphorylation and activation of STATs, which migrate to the nucleus and interact with specific DNA-binding sites [36,37]. Different proteins of the JAK and STAT family are involved in different tasks such as immunomodulation, transcription, and tumorigenesis. In gliomas, JAK-1 and STAT-3 appear to be the major proteins that are altered in the JAK/STAT pathway. The expression of these two proteins was higher in gliomas than in normal brain tissue, and it was also confirmed that marked expression was more frequent in WHO grade 4 glioblastomas (47%) than in WHO grades 2 and 3 gliomas (22% and 26%) [37]. There is currently a lot of interest in finding cofactors and inhibitors for this signaling pathway. An example of a JAK activator is the Runt-related transcription factor 1 (Runx1), which is an antagonist of JAK suppressors of cytokine signaling 3 and 4 (SOCS3 and SOCS4). Runx1 inhibits the activation of SOCS3 and SOCS4, leading to the activation of the JAK/STAT pathway [38]. VPS25 is a member of the endosomal sorting complex required for transport (ESCRT) and is involved in many functions such as the degradation of ubiquitinated proteins, the budding of retroviruses, and cell cycle control. This protein was also upregulated in glioma tissue and correlated with poor prognosis in glioma patients [39]. One inhibitor of the JAK/STAT pathway is ruxolitinib, which is known to have an antitumor effect in various tumor types such as colon or ovarian cancer. In an in vitro study, it was shown to inhibit the phosphorylation of JAK/STAT proteins, resulting in reduced growth and invasiveness of U87 glioblastoma cells [40]. The inhibition of JAK/STAT signaling by the NOTCH pathway has been demonstrated in *Drosophila*, and it remains to be determined whether this also applies to other species [41]. The NOTCH signaling pathway is involved in stem cell maintenance and cell survival, vascular maturation, and tumor recurrence and progression after treatment. Signaling activation is triggered by the interaction of one of the four receptors NOTCH1-4 with the ligands Jagged1-2 or Delta-like 1-4, followed by proteolytic cleavages of the receptor by ADAM metalloproteinases or by γ-cleavage [42]. In gliomas, NOTCH receptor expression is increased and correlates with the degree of malignancy, whereas it is only slightly expressed in normal tissue [43]. This signaling pathway is unique to gliomas for two reasons: First, it is not a pathway of soluble molecules, but a cell-to-cell interaction with transmembrane ligands that bind and activate a transmembrane receptor of a neighboring cell. Secondly, NOTCH signaling is a double-edged sword when it comes to exploiting this pathway for therapeutic options, as its down-regulation has implications for reduced tumor growth, but on the other hand, reduced NOTCH signaling may promote aberrant tumor vessel formation and thus promote tumor growth [36,42,43]. There is a need for further clarification here, and the question even arises as to whether targeting NOTCH signaling has different effects at different stages of tumor progression [42]. Of growing interest in glioma research is the WNT signaling pathway, which is known to play a role in tumor growth and even angiogenesis in several tumor types. Although there is currently no firm evidence for the involvement of the WNT pathway in glioma angiogenesis, there are several observations demonstrating a significant role of this pathway in glioma progression and invasiveness, as well as resistance to temozolomide chemotherapy [44,45,46,47]. One example is the study of fragile X mental retardation protein (FMRP). This is a protein that is expressed primarily in the brain and is important for normal cognitive development. FMRP was found to be expressed more abundantly in human glioblastomas, which inversely correlated with patient survival. The main effect of experimentally reducing FMRP expression was the downregulation of both the canonical and non-canonical WNT pathways, suggesting that the malignant properties of glioma cells are influenced by the regulation of the WNT pathway [48]. The hippo signaling pathway is physiologically involved in the regulation of cell proliferation in the context of tissue development and regeneration. Its major kinases are the tumor suppressors mammalian STE20-like protein kinase 1 and 2 (MST1/2) and large tumor suppressor 1 and 2 (LATS1/2), as well as the two protooncogenes Yes-associated protein 1 (YAP1) and its orthologue, transcriptional co-activator with PDZ-binding motif (TAZ). In the physiologically activated hippo pathway, phosphorylation and activation of MST1 and MST2 leads to activation and phosphorylation of LATS1 and LATS2, which in turn leads to phosphorylation and consequent inactivation and degradation of YAP1/TAZ in the cytoplasm. In the inactive hippo pathway, inactivation of YAP/TAZ does not occur, and both proteins enter the nucleus and form complexes with TEA domain (TEAD) family transcription factors, leading to increased gene expression, proliferation, and migration [49,50]. Involvement of the inactivated hippo pathway due to decreased expression of MST1/2 or LATS1/2 or even due to the overactivation of YAP1 and TAZ has been confirmed in various tumor types, such as colorectal and prostate cancer and even in astrocytomas and glioblastomas [50]. In a series of 88 tumors, the percentage of tumors with downregulation of LATS1 and LATS2 due to promoter hypermethylation increased significantly from 48%/52% in grade 2 astrocytomas to 65%/70% in grade 3 astrocytomas and to 73%/82% in grade 4 glioblastomas [51]. The degree of overexpression of YAP1 has also been shown to correlate with the degree of tumor progression in glioblastomas [49,50]. The hippo signaling pathway can be activated or inactivated by different molecular mechanisms depending on the cellular context. An important activator is neurofibromin 2 (Merlin), and an important inactivator is the S100 protein S100A16, which leads to decreased LATS1 expression and the promotion of glioma progression [49,52]. As the hippo signaling pathway can be modulated by several other signaling pathways, such as MAPK, WNT, NOTCH, and by different tyrosine kinase receptors (RTKs), the hippo signaling pathway can be considered a prime example of the distinct interconnectivity of all molecular signaling pathways in the mammalian cell, including glioma cells [49,50].

## 4. Angiogenic and Apoptotic Pathways

The angiogenic pathways in tumors are highly interconnected, as the activation of factors also leads to the activation of other downstream signaling pathways that promote tumorigenesis. The most important pro-angiogenic factor is vascular endothelial growth factor receptor (VEGFR), but other factors also contribute to tumor angiogenesis, such as fibroblast growth factor receptor (FGFR), angiopoietins 1 and 2 (Ang1 and Ang2), erythropoietin (EP), transforming growth factor beta (TGF-β), and matrix metalloproteinases (MMPs) [4]. The increased expression of some of these factors is supported by hypoxia-inducible factor 1 (HIF1), which in turn is stimulated by several mechanisms, including the overexpression of EGFR, mutations of tumor suppressors such as p53 or PTEN, and hypoxia, which is an important factor in the activation of angiogenic signaling pathways. HIF1 then also induces the release of growth factors, matrix components, and adhesion molecules [4,53]. Some pro-angiogenic factors can stimulate angiogenesis independently, such as EGFR and EGFRvIII [54] and hepatocyte growth factor/scatter factor (HGF/SF), which can induce angiogenesis independently of VEGF [4]. On the other hand, simultaneous targeting of different factors could improve the anti-angiogenic effect, as shown by the combined experimental targeting of VEGF and Ang2, leading to longer animal survival [55]. Currently, the main interest in angiogenic pathways is to find fixed points in these metabolic pathways that may be mediated by specific proteins. The transmembrane protein vasorin (VASN) is involved in vascular injury repair and development. It promotes angiogenesis in vitro and in vivo by activating STAT3 and inhibiting NOTCH signaling. Patients with glioblastomas who had high expression of VASN had shorter overall survival, and the expression level also correlated with microvessel density in the tumors [56]. Ras Homolog Family Member J (RhoJ) is expressed in endothelial cells and promotes endothelial cell migration and tumor angiogenesis. An in vitro study suggests that it may also play a role in glioblastoma angiogenesis [57], which is also true for the homeodomain transcription factor paired-related homeobox 1 (Prrx1) and for a GTPase named ADP-ribosylation factor-like protein 13B (ARL13B) [58,59]. The binding of CXC-motif chemokine receptor 2 (CXCR2) by its ligand CXCL2 provides an alternative signaling pathway to induce angiogenesis in gliomas via activation of immune host cells and independently of VEGF signaling [60]. It has been shown that blocking CXCR2 in an experimental glioma model resulted in a reduction in tumor volume by up to 50%, along with a reduction in invasiveness and lower tumor vessel density [61]. Another interesting topic is the possibility of targeting molecules involved in both the process of tumor angiogenesis and apoptosis in gliomas. Hypoxia-inducible factor 1 (HIF1) plays a central role in this context by activating pro-apoptotic proteins such as BNIP3 and NIX and even stimulating angiogenesis, extracellular matrix metabolism, and cell proliferation [62]. Cabazitaxel is a semisynthetic taxane used to treat prostate cancer and has the ability to be absorbed through the endothelia of the blood–brain barrier. In a human glioma cell model, cabazitaxel disrupts cytoskeletal F-actin fibers and induces tumor cell apoptosis. Moreover, tumor cell growth and even tumor-induced angiogenesis were inhibited. Remarkably, this anti-angiogenic effect was limited to tumor vessels and could not be reproduced in vessels from normal brain tissue [63]. Survivin is an inhibitor of apoptosis, and simultaneous stimulation of tumor growth, angiogenesis of tumor vessels, and inhibition of tumor cell apoptosis have been confirmed in several tumor types, including gliomas [64]. In addition to the known involvement of HIF1 in both angiogenesis and apoptosis, their precise molecular interrelationship is still only partially understood. Deregulation of apoptotic signaling pathways plays an important role in gliomas and involves both types of apoptosis, the extrinsic (“death receptor”) and intrinsic (“mitochondrial”) pathways. The extrinsic pathway is mediated by the activation of tumor necrosis factor receptors (TNFRs) in the cell membrane by their ligands, such as tumor necrosis factor-related apoptosis-inducing ligand (TRAIL) or TNF receptor superfamily member 6 (FAS). Activation of TNFRs leads to the cleavage of procaspase-8 into caspase-8, which in turn cleaves effector caspase-3. The mitochondrial apoptotic pathway is independent of cell membrane receptors and relies on the interplay of anti-apoptotic proteins such as BCL-2 and proapoptotic proteins such as BAK and BAX, which promote the release of cytochrome-c into the cytosol. This in turn leads to the cleavage of procaspase-9 to caspase-9 and further to the cleavage and activation of effector caspase-3 [5,65,66]. TP53 is involved in both the extrinsic and intrinsic apoptotic signaling pathways. It can increase the expression of FAS by transcription, triggering the activation of the extrinsic pathway, and it can activate the pro-apoptotic protein BAX within the intrinsic pathway, inhibiting the activity of BCL-2 [65,67]. Another pathway of TP53 involvement in apoptosis is the transactivation of PTEN by TP53, with subsequent deactivation of AKT and activation of caspase-9. In turn, activation of AKT leads to inhibition of caspase-9 activation and inactivation of pro-apoptotic proteins [65]. Deregulation of apoptosis has been well studied in glioblastomas and is reflected by significant downregulation of proapoptotic proteins such as BAX, BAK, and procaspase-9 and upregulation of anti-apoptotic proteins such as BCL-2 [65,68,69]. Even a correlation of proteins with patient survival was confirmed in a cohort of 188 patients showing a positive correlation between the expression of BAX and survival [70] and in a cohort of 97 patients with a positive correlation between the expression of cleaved caspase-8 and survival [71]. An indirect link to the inhibition of apoptosis in gliomas is the alternative lengthening of telomeres (ALT) due to mutations of the *gene for telomerase reverse transcriptase (TERT)* that lead to TERT overactivation, thereby preventing tumor cells from apoptosis. TERT mutations are found in many glioblastomas, whereas mutations of the *X-linked gene of α-thalassemia (ATRX)*, leading to inactivation of the ATRX protein and induction of ALT, are found in many IDH-mutated astrocytomas. Of note, TERT mutations and ATRX mutations are mutually exclusive in gliomas, and the exact mechanism of how ATRX mutations, in particular, induce ALT in gliomas is still under investigation [72,73,74,75]. Apoptosis in gliomas is still a topic with many unresolved issues, and a major problem is finding the reason for the frequent TRAIL resistance of glioma tumor cells. Progress in overcoming TRAIL resistance in vitro has been made by increasing the expression of proapoptotic proteins by silencing the histone demethylase KDM2B [76] and even by silencing eukaryotic initiation factor 5B (eIF5B), an oncogenic stress-related protein [77]. Another interesting topic is the interrelationship between apoptotic signaling pathways and those of autophagy. Autophagy, closely linked to the PI3K/AKT/PTEN and mTOR pathways, represents an alternative mechanism for cell death besides apoptosis, with many alternative modes of activation. Central proteins in these cascades are ULK1/2, Beclin-1, and the so-called *UV irradiation resistance-associated tumor-suppressor gene (UVRAG)* [65]. A major challenge in current research is that autophagy can promote or suppress tumor progression depending on the cellular context and the regulation or deregulation of tumor-suppressor genes. Moreover, autophagy can trigger or even inhibit the molecular cascade of apoptosis, confirming the need for further studies on the interrelationship between apoptosis and autophagy [65]. 

## 5. Metabolic Pathways

An important alteration in metabolism, especially in highly proliferating gliomas, is that tumor cells meet their energy requirements by glycolysis instead of oxidative phosphorylation. This phenomenon is known as the Warburg effect and results in a high rate of glycolysis in proliferating tumor cells, including an overproduction of lactate. In the absence of significant efflux, increased cellular lactate inhibits glioma invasion. Therefore, accelerated lactate efflux is necessary to promote tumor cell invasiveness [1]. This lactate efflux is carried out by transmembrane monocarboxylate transporters (MCTs), with MCT1 and MCT4 being the main transporters in glioma cells. Both MCTs are regulated by co-expression with their chaperone CD147 and contribute to the maintenance of glycolytic metabolism and tumor cell survival. In vitro inhibition of MTCs by alpha-cyano-4-hydroxycinnamate (CHC) resulted in a decrease in tumor cell metabolism and invasion and was able to induce tumor cell death, which was further enhanced by a synergistic effect when CHC was combined with temozolomide [2,78]. A prior step in glycolysis is the conversion of phosphoenolpyruvate to pyruvate, which is catalyzed by pyruvate kinase. Alterations in pyruvate kinase type 2 (PKM2) have been found in various cancers, e.g., overexpression in glioblastomas, whereas no overexpression of PKM2 was found in normal brain tissue [79]. EGFR activation has been shown to provide transport of PKM2 into the nucleus and its binding to β-catenin. The level of expression of PKM2 in the nucleus correlated with the malignancy grade of gliomas, confirming a non-metabolic role of PKM2 in glioma progression due to its binding to β-catenin as a member of the WNT pathway [80]. In addition to glycolysis, glutamine metabolism is another important metabolic pathway in tumors that serves as a source of amino acid and nucleotide synthesis. PET imaging in vivo with the glutamine analog tracer 18F-FGln confirmed a high uptake in gliomas, particularly those showing marked progression. This uptake declined after chemo/radiotherapy, and there was no uptake in normal brain areas or in areas of neuroinflammation [81]. In the cytoplasm, glutamine is converted to glutamate by glutaminase isoenzymes 1 and 2 (GLS1 and GLS2), and in malignant gliomas, GLS2 has been reported to be downregulated while GLS1 is expressed [82,83]. Experimental blocking of glutaminases in combination with a calorie-restricted ketogenic diet reversed tumor symptoms and improved overall survival in *mice*, confirming the importance of glucose and glutamine pathways in the growth of malignant gliomas and providing a potential therapeutic strategy [84]. In IDH-mutated gliomas, particularly diffuse astrocytomas and oligodendrogliomas, isocitrate dehydrogenase (IDH) metabolism is altered with the production of the oncometabolite 2-hydroxyglutarate (2-HG). IDH mutations can occur in various cancers, such as acute myeloid leukemia, melanoma, and cholangiocarcinoma. IDH mutations in gliomas mostly relate to isotype 1 (IDH-1) and less frequently to isotype 2 (IDH-2) [1]. Under physiological conditions, IDH mediates the oxidative decarboxylation of isocitrate to form α-ketoglutarate (α-KG) or 2-oxoglutarate (2-OG) as part of the citric acid cycle. The chemical structure of 2-HG is similar to that of α-KG, except that the carbonyl group at the C2 position is replaced by a hydroxyl group. Therefore, 2-HG interferes with enzymes using α- KG as a substrate, competitively inhibiting the activity of α-KG [1,85]. Disruption of the normal citric acid cycle leads to impaired recycling of the antioxidant glutathione, resulting in the accumulation of reactive oxygen species (ROS) and increased oxidative stress. Although IDH-mutated glioma cells lack TERT mutations, they are able to reactivate the telomerase TERT and stabilize their telomeres in association with increased expression of c-MYC target genes, including TERT [86]. Another consequence of deregulated IDH metabolism in IDH-mutated glioma cells is the downregulation of leukocyte chemotaxis, leading to a significant reduction of most immune cells (macrophages, microglia, CD8+ CD4+, B cells, and dendritic cells). It is postulated that 2-HG is responsible for this effect, and further studies on the role of 2-HG in tumor stroma and the clinical significance of this finding are planned [87,88]. One known effect of 2-HG is to inhibit the activity of Ten-eleven translocation enzymes (TETs), which play a critical role in regulating DNA methylation and gene expression. This action of 2-HG leads to genome-wide DNA hypermethylation, the so-called CpG island methylation phenotype in IDH-mutated gliomas (G-CIMP), which are known to have a better prognosis than malignant gliomas with IDH wild-type [85]. However, approximately 10% of gliomas with G-CIMP have only a low degree of hypermethylation, termed G-CIMP-low. These tumors exhibit more aggressive behavior with a significantly worse patient outcome. G-CIMP-low tumors are also thought to have a specific epigenetic signature, but the exact molecular and metabolic mechanisms of the development of these tumors, which arise either de novo or from G-CIMP-high tumors, remain to be further explored [85,89]. Tumor development and progression of IDH wild-type glioblastomas are highly dependent on the overexpression of RTKs involving major signaling pathways such as the EGFR/PI3K/AKT/PTEN pathway but also on the involvement of metabolic pathways. EGFRvIII is a common mutation of the extracellular domain of the *EGFR gene* with deletion of exons 2-7, present in the majority of glioblastomas with EGFR amplification. An important effect of EGFRvIII is the activation of heterogeneous nuclear ribonucleoprotein A1 (hnRNP-A1), which induces the formation of the protein DeltaMax by splicing the Myc-interacting protein Max. DeltaMax is a potent promoter of glycolysis and tumor growth in vivo, and a study of 131 glioblastoma patients showed a significant correlation between increased expression of hnRNP-A1 and glycolytic genes with shorter overall survival [90]. In addition to glycolysis, the biosynthesis and presence of fatty acids and cholesterol are also important for basic structures such as cell membranes in proliferating glioma cells. The β-oxidation of fatty acids and the continuous release of stored fatty acids by lipid droplets have been shown to play a central role in the cellular survival of glioblastoma cells [91,92]. Moreover, de novo lipogenesis of fatty acids is an important metabolic process, as evidenced by the overexpression of the key enzyme fatty acid synthase (FAS) in glioblastoma cells. Overactivation of TERT due to TERT mutation in glioblastoma cells increased FAS levels and lipid accumulation, which in turn were reduced by a TERT inhibitor [93]. The importance of cholesterol synthesis for tumor growth was confirmed by the experimental application of the liver X-receptor 623 (LXR-623), which is a ligand for oxysterols that is able to lower cellular cholesterol levels by promoting cellular sterol efflux. This effect of LXR-623 led to cell death of glioma cells in vitro and a significantly prolonged survival of the animals in a patient-specific GB model [94]. 

## 6. Epigenetic Processes

**Figure 1:**
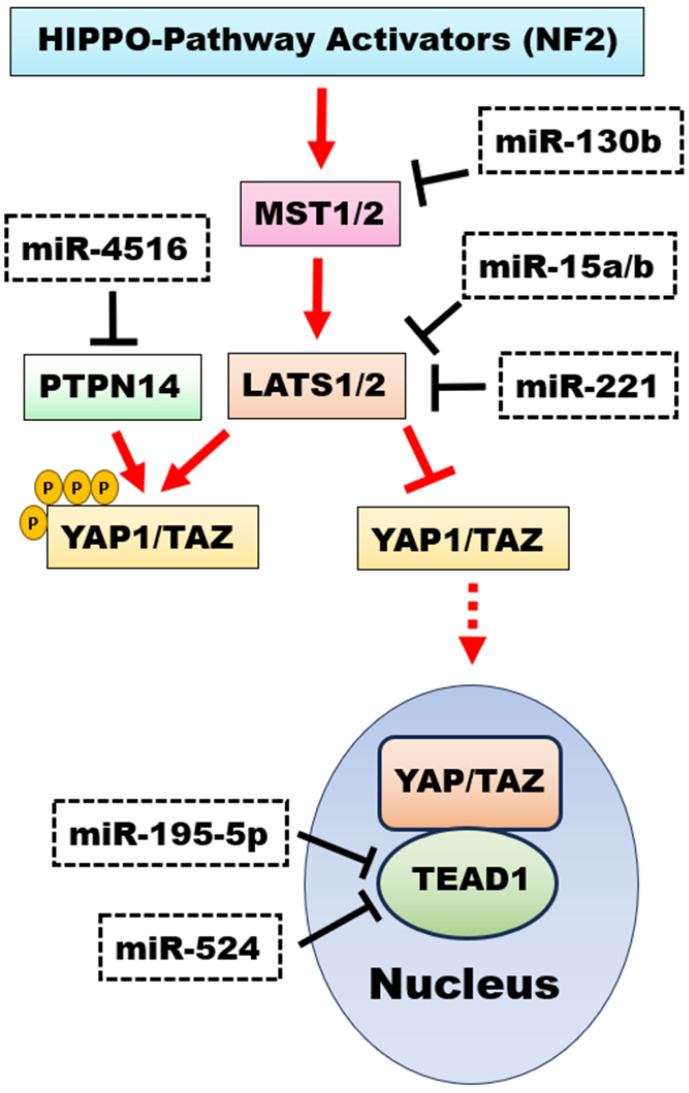
Influence of microRNAs on the hippo signaling pathway in gliomas. Major tumor suppressors of the hippo pathway, mammalian STE20-like protein kinases 1 and 2 (MST1/2), and major tumor suppressors 1 and 2 (LATS1/2) can be downregulated by the microRNAs miR-130b, miR-15a/b, and miR-221 [121,122,123]. The tumor-suppressor protein tyrosine phosphatase non-receptor type 14 (PTPN14) can be downregulated by miR-4516 [124]. In the nucleus, the transcription factor TEA domain member 1 (TEAD1) forms a complex with the active (non-phosphorylated) proto-oncogene Yes-associated protein 1 (YAP1) and the transcription co-activator with PDZ-binding motif (TAZ), thereby promoting transcription activity, proliferation, and cell survival. TEAD1 can be downregulated by the microRNAs miR-195-5p and miR-524 [125,126].

**Figure 2:**
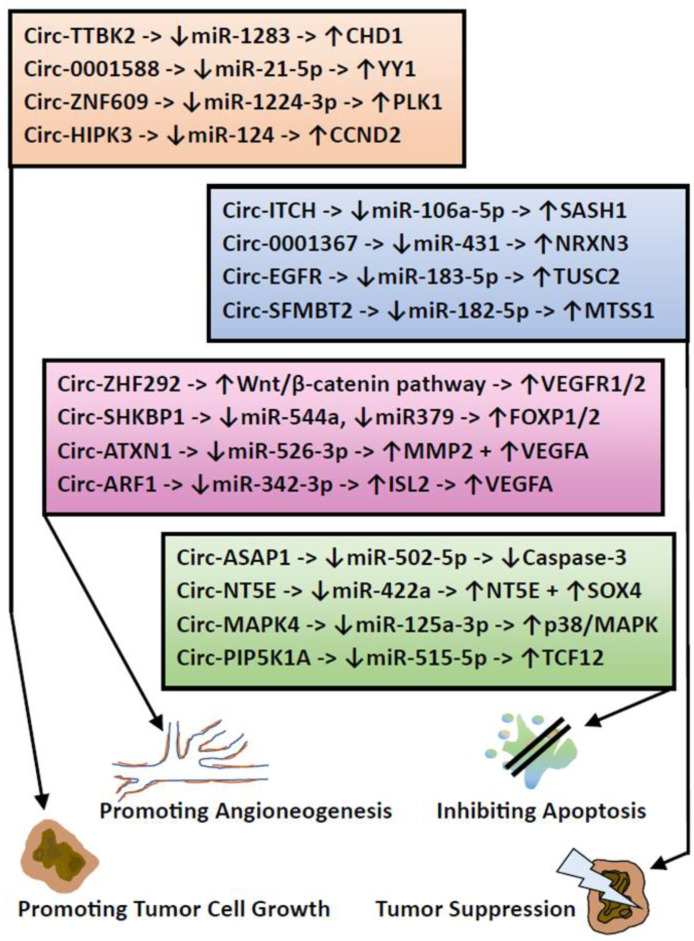
List of circular RNAs (Circ-RNAs) in gliomas involved in tumor progression [129,130,131,132], tumor suppression [141,142,143,144], angioneogenesis [133,134,135,136], and apoptosis inhibition [137,138,139,140]. For each Circ-RNA, its main targets are listed, being upregulated (↑) or downregulated (↓) by the Circ-RNA.

The term epigenetics refers to molecular processes involved in the regulation or alteration of gene expression that are not associated with a change in gene sequence. The three most important epigenetic processes are methylation, histone alterations, and regulation by so-called “non-coding RNA molecules”. The molecular basis for the methylation of DNA or other biomolecules are methyltransferases classified according to the ‘Enzyme Nomenclature Database’ of subclass 2.1.1 [95]. DNA methyltransferases allow the transfer of a methyl group to a certain position within a nucleobase, while some methyltransferases (demethylases) are able to remove methyl groups from certain positions [96,97]. In human DNA, most methylation sites are CpG sites with a methyl group at position 5 of the cytosine. The function and significance of many methyltransferases in *Homo sapiens* are certainly still largely unknown. So far, the focus has been on investigating the DNA methylation profile in diseases and even gliomas. In gliomas, DNA methylome analysis can be performed using bisulfide PCR or even novel strategies, such as nanopore sequencing, but most clinical applications are still based on chip arrays with a readout of more than 800,000 methylation sites. A statistical comparison of the results with thousands of reference samples from the “Classifier of Molecular Neuropathology” [98] contributes to the diagnostic classification of the individual case in the clinical routine [99]. Although mutations cannot be detected directly by methylome analysis, the indirect detection of important changes such as 1p/19q codeletions in oligodendrogliomas, IDH mutations, or even methylation of O6-methylguanine-DNA-methyltransferase (MGMT) can be performed due to the resulting changes in methylation patterns [31,99]. The analysis of MGMT methylation is of paramount importance for gliomas, as it occurs in more than half of IDH-mutated astrocytomas and in about half of IDH wild-type glioblastomas. Under physiological conditions, non-methylated and thus active MGMT removes oncogenic alkyl groups from the O6 position of guanine. In gliomas with MGMT-promoted methylation, MGMT is inactive and does not affect alkylating agents such as temozolomide. Therefore, MGMT methylation in gliomas is a favorable prognostic finding, independent of therapeutic modalities such as the use of alkylating agents [96,100]. Several observations underline the continuing progress in the understanding of DNA methylation, e.g., regarding the previous rule that methylation of a gene in its promoter region generally leads to transcriptional suppression. This rule is no longer invariable, as important exceptions have been described, such as the *forkhead box A2 gene (FOXA2)*, which plays a role in prostate cancer and whose promoter methylation leads to activation of the *FOXA2 gene* expression [100]. It is also worthwhile to find similar exceptions to this rule in the case of glioma pathology. Another new finding relates to N6-methyladenosine, which is especially enriched in heterochromatin and appears to be involved in the pathobiology of glioblastomas, which represents an important relationship between methylation and histone alterations [101,102]. Temozolomide treatment upregulates methyltransferase METTL3, which potentiates N6-methyladenosine-associated epigenetic alterations of histones such as histone 3 acetylation at position 27 (H3K27ac). These modifications promote temozolomide resistance, whereas experimental METTL3 targeting in combination with temozolomide suppresses glioma growth [101]. For other demethylases of N6-methyladenosine such as the AlkB homologues 1 and 5 (ALKBH1 and ALKBH5) in glioblastomas, marked overexpression along with concomitant histone changes such as triple methylation of histone 3 at position 9 (H3K9me3) has been reported. This led to the promotion of tumor growth in vitro and in vivo, with the role of these demethylases as potential biomarkers and therapeutic targets for glioblastomas being addressed [102,103]. The role and interaction of histone acetyltransferases (HATs) and histone deacetylases (HDACs) in tumors is complex and not yet fully understood. Although HATs and HADCs are opposing regulators of histone acetylation, the overexpression of HATs and even HADCs has been shown to promote glioma growth. Histone acetyl transferase KAT6A acetylates lysine of histone H3 at position 23, leading to an overactivation of the PI3K/AKT pathway and an increased glioblastoma tumor growth in vitro [104]. Overexpression of several HADCs, such as HDAC 1, 6, and 9, was confirmed in glioblastomas, and the knockdown of these HADCs showed reduced cell migration, reduced tumor growth, and even induction of apoptosis [105,106,107]. Experimental attempts have been made to use HDACs such as panobinostat as a therapeutic target against several cancers, especially gliomas, whereby overcoming the blood–brain barrier (BBB) by alternative routes of intracerebral administration is a major challenge, also with regard to possible side effects such as tissue damage and necrosis [108,109,110,111]. A mutation in one of the histone-3 sequence variants H3.1, H3.2, or H3.3, which leads to a widespread loss of trimethylated H3K27me3 in midline glioma, is diagnostic for the so-called diffuse midline glioma (DMG). The combination of a loss of trimethylated H3K27me3 with an aberrated overexpression of the zest homolog inhibitor protein (EZHIP) or an EGFR amplification of a midline glioma is also sufficient for the diagnosis of DMG [112]. This histone H3K27 alteration has reached diagnostic significance since the WHO classification of CNS tumors of 2016 [113], but it is no longer specific to DMG, according to the fifth edition of 2021, since in rare cases, H3K27 alterations can also occur in other gliomas, such as pilocytic astrocytoma, ganglioglioma, and even in tumors outside the midline [114,115]. Another mutation, H3G34, is specific to the so-called diffuse hemispheric glioma, the H3G34-mutant. Both tumor types differ in their midline location, including the thalami (H3K27-altered) or within the hemispheres (H3G34-mutated), but both tumors are WHO grade 4 tumors with poor prognosis, regardless of their histopathological or imaging phenotype [115]. The exact molecular mechanisms leading to tumor development and progression are still being investigated for both tumor types, which are characterized by specific histone alterations [112,116]. Non-coding RNA molecules (ncRNAs) represent the third major complex of epigenetic regulation. The majority of the entire transcriptome consists of ncRNAs, while protein-coding RNAs make up less than 5% of the transcriptome [117,118]. The three main types of ncRNAs are microRNAs (miRNAs) with a length of about 18–22 nucleotides (nt), long non-coding RNAs (lncRNAs) with a length of >200 nt, and circular RNAs (circRNAs), characterized by a covalent circular bond without 5′-3′ polarity [117,119,120]. These ncRNAs interact with each other and play an important role in regulating differentiation, apoptosis, cell survival, and proliferation. The main function of miRNAs is the negative regulation of gene expression by interaction with the 3′ untranslated region of their target [117]. Since these targets can be (proto-)oncogenes or tumor suppressors, the corresponding miRNA itself can act as an oncogene or tumor suppressor. Each molecular pathway in gliomas is subject to regulation by miRNAs. An important example is the hippo signaling pathway, since it is already crosslinked to a significant extent with other molecular pathways [49,50,121]. As shown in Figure 1, different miRNAs act as oncogenes by downregulating important tumor suppressors of the hippo pathway [121,122,123,124], while other miRNAs may act as tumor suppressors [125,126] (Figure 1). Long non-coding RNAs are primarily competitors of miRNAs, as they are able to prevent interaction of miRNAs with their targets by means of sponging. One example is the overexpression of the lncRNA LINC00473 in glioma tissue. This in turn leads to a downregulation of miRNA-195-5p with a reduction of its activity as a tumor suppressor [118,125,127]. Other factors, such as EGFR overexpression or EGFRvIII mutations leading to the downregulation of miRNA-524, also have the ability to suppress miRNA activity in gliomas. This miRNA-524 acts as a tumor suppressor by downregulating the complex TEAD1 [126] (Figure 1). Like lncRNAs, circular RNAs (circRNAs) primarily act as competitors of miRNAs by sponging them, but some other functions are addressed, such as a direct influence on transcription and splicing. Even the coding of functional proteins has been confirmed for individual ncRNAs, which relativizes the term “non-coding” [6,128]. In gliomas, various circRNAs have been described that promote tumor progression [129,130,131,132], promote angiogenesis [133,134,135,136], inhibit apoptosis [137,138,139,140], and even circRNAs that act as tumor suppressors [141,142,143,144] (Figure 2). The function of these circRNAs is to inhibit their target miRNAs, leading to the overexpression of oncogenes such as transcription factor Yin Yang 1 (YY1) [130] or to the upregulation of angiogenic factors such as vascular endothelial growth factor A (VEGFA) [136]. Other possible consequences of the activity of circRNAs are the downregulation of apoptotic effectors such as caspase 3 [137] or the upregulation of tumor suppressors such as the presynaptic adhesion molecule neurexin 3 (NRXN3), which inhibits glioma growth in vivo [142] (Figure 2). As expected, circRNAs with a tumor-suppressor function were downregulated in glioma cells [141,142,143,144]. 

## 7. Extracellular Vesicles

It is well known that membrane-bound particles of different sizes can be released from normal cells and tumor cells into the extracellular space. According to their size and their different origins, several terms for these particles have been used, but as an umbrella term, the expression “extracellular vesicles” (EVs) is widely accepted [145,146,147]. They may contain DNA, mRNA, ncRNAs such as miRNAs, and proteins, and it is known that tumor cells express more EVs than normal cells [148,149]. Currently, a general distinction is made between two important types of EVs: (1) exosomes, originating from the endosomal system, and (2) microvesicles (also called ‘ectosomes’), originating directly from outward budding or pinching of the cellular plasma membrane. According to different study groups, the size range of exosomes is reported to be approximately 30–200 nm, and the size range of microvesicles is from 100 nm up to 1 µm [145,147,148,150]. Due to the overlapping size range and the ongoing search for standardized isolation and purification methods for EVs, a differentiation between exosomes and ectosomes may be difficult so far [148,149]. Other types of EVs are the so-called “apoptotic bodies”, and even other types of EVs have been defined in the literature with terms such as “oncosomes” or “macrovesicles” [146,150]. However, regardless of ongoing efforts to nomenclature and optimize laboratory protocols and methods, EVs generally represent an essential transport and communication system between cells, the extracellular environment, and body fluids. In neurodegenerative diseases, EVs contain pathological proteins such as β-amyloid or α-synuclein [145,151]. In gliomas, there are indications that the content of tumor cell-based EVs reflects the phenotypic and thus molecular signature of the respective glioma cells to a large extent [152]. Many studies have confirmed the contribution of EVs to glioma angiogenesis, migration and proliferation, invasiveness, activation of molecular signaling and therapeutic resistance [149]. An important aspect of the current literature on EVs in gliomas is the frequent reports of non-coding RNAs (ncRNAs) delivered by EVs. An important example is the promotion of glioma angiogenesis by the secretion of exosomes enriched with the long non-coding RNA Lnc-POU3F3 [153]. Even a promotion of glioma angiogenesis has been reported by exosomal stimulation of the expression of miR-21, VEGF, and VEGFR2 in endothelial cells [154]. As expected, with exosomal administration of the metalloproteinases MMP2 and MMP9, an increased progression of glioma growth with increased invasiveness was reported [149]. EV-packed miR-30b-3p has been shown to mediate decreased apoptosis of glioblastoma cells in association with increased proliferation and resistance to temozolomide [155]. Another important aspect of current EV research is the attempt to use EVs as a therapeutic delivery system, such as the experimental improvement of EV penetration through the blood–brain barrier by the combined administration of doxorubicin with angiopep-2 and a transactivator of transcription (TAT) with pronounced transcytosis capacity. This combination resulted in a 2-fold increase in the survival time of the animals with only minor side effects [156]. Even the EV delivery of miR-124 to human glioma cells in vitro led to reduced tumor growth with significant downregulation of cytokine expression [157]. Taken together, a variety of different molecules, especially ncRNAs and proteins, can be contained and transported by EVs in gliomas, confirming the central role of EVs in molecular signaling processes and pathways in these tumors. Further refinement and improvement of purification methods for EVs are strongly discussed in the literature in order to maximize the potential of EVs as a diagnostic target and as a potential therapeutic delivery system [146,148,149]. 

## 8. Discussion

The distinct interconnectivity of molecular cellular signaling pathways in terms of their involvement in tumorigenesis and glioma growth due to dysregulation and alterations is an important recent finding in glioma research. The second most important discovery in recent years is the recognition of the significant impact of the group of non-coding RNAs (ncRNAs) on molecular pathobiology in tumors, as it appears that ncRNAs, especially circular RNAs (circRNAs), have an overriding influence on the regulation of molecular signaling pathways in gliomas. Most circRNAs are formed by circularization of exon sequences, and only a few circRNAs are formed from introns [144]. Most circRNAs are localized in the cytoplasm and some in the nucleus, but of growing interest is their transport through extracellular vesicles (EVs) that are secreted by various cell types [144,158]. EVs have already gained considerable interest in tumor research, and in relation to gliomas, EVs have been shown to contribute to tumor growth in many different ways, mainly by transporting proteins and ncRNAs [152,153,154,155,156,157]. This is also true for circRNAs that are contained in extracellular vesicles such as exosomes, as these circRNAs appear to exert important functions with respect to their own regulatory role within the specific tumor type and signaling pathway. Together with advances in EV sampling methods, circRNAs transported by EVs may serve as important biomarkers for gliomas [158]. Indeed, the competitive relationship between the different types of ncRNAs has been confirmed, as had the direct influence of individual ncRNAs on signaling pathways such as an EGFR-independent activation of epidermal growth factor (EGF) by a circular RNA called “circ-E-Cad” overexpressed in glioblastomas [159]. The possibility of reverse signaling protein effects on ncRNAs can also be considered certain, such as repression of miRNA-524 by EGFR overexpression or EGFRvIII mutation in glioblastomas, thereby inhibiting the tumor-suppressor activity of this miRNA [126]. The highly interconnected functionality of ncRNAs highlights the diversity of activation and regulation of molecular signaling pathways, especially when additionally considering the diversity of tyrosine kinase receptors and other currently studied receptors such as integrins and even chemokines [6,21,23,24]. In addition to the known involvement of signaling pathways such as PI3K/AKT/PTEN, the TP53 pathway or the RB1 pathway in glioma growth, the importance of other signaling pathways in this regard is increasingly being explored. An important example is the investigation of activators of the JAK/Stat pathway such as Runx1 and VPS25, which are overexpressed in glioma tissues and thus serve as potential therapeutic targets [38,39]. Currently, research on the NOTCH pathway is particularly challenging, as its downregulation may have negative effects on gliomas by impairing tumor growth but also tumor-promoting effects by promoting angiogenesis [36,42,43]. The role of the WNT pathway is also becoming increasingly important in glioma research, and evidence for its involvement in glioma growth is the tumor-promoting effect of the RNA-binding protein FMRP, which is overexpressed in glioblastomas. As confirmed by transcriptome analyses, the WNT pathway is the most enriched pathway among published FMRP target genes [48]. This relationship was further confirmed by the downregulation of the WNT pathway due to an experimental reduction in FMRP expression, arguing for a significant role of the WNT pathway in the pathophysiology of gliomas [44,48]. The same is true for the hippo pathway, which in its physiologically active form is a tumor-suppressor pathway. In gliomas, its inactivation leads to an active state of the oncogenes YAP/TAZ that promotes glioma growth [49,50]. 

Regarding tumor angiogenesis in gliomas, the search for targeted fixed points is currently an important goal, and in particular, experimental progress has been made by simultaneously targeting VEGF and Ang2 in a mouse model with prolonged animal survival [55]. There are many other proteins with pro-angiogenic activity currently under investigation in gliomas, such as the transmembrane protein vasorin [56], Ras homolog family member 1 (Rho1) [57], and chemokine receptor 2 (CXCR2), along with its ligand CXCL2 [60]. Of particular interest are proteins involved in both promoting angiogenesis and inhibiting apoptosis. An example is forkhead box M1 (FOX1) and its target survivin, which are upregulated in gliomas, and their high expression correlates with poor patient prognosis [64,160]. In a temozolomide (TMZ)-insensitive cell line and in a mouse model of TMZ-resistant high-grade gliomas, blocking the FOX1/survivin axis with the protease inhibitor bortezomib resulted in decreased tumor growth with increased apoptotic activity. Even a synergistic effect of bortezomib and TMZ administration was demonstrated with sensitization of glioma cells to TMZ treatment in vivo and in vitro with reduced proliferation and tumor growth [64,160]. Overcoming therapy resistance also involves TRAIL resistance of glioma tumor cells, and progress has been made even in this regard by sensitizing tumor cells to TRAIL-induced apoptosis through inhibition of KDM2B and eIF5B. Both proteins are upregulated in various tumor types, and there is a need to further explore their precise roles in tumors [76,77]. Outlining all the signaling pathways and key molecular points potentially responsible for therapy resistance in gliomas in vitro or in vivo is a separate and comprehensive topic that is far beyond the scope of the present review. A review of this topic must also include the interactions between the intrinsic properties of glioma cells and their microenvironment, which contains a variety of non-neoplastic immune cells that strongly influence glioma growth and response to treatment [147,161]. It is important to recognize that many individual mechanisms, such as resistance to TRAIL, can contribute to treatment resistance, and even TMZ resistance has been shown to occur independently of non-methylated MGMT activity [162]. One reason for therapy resistance of gliomas may be defective DNA mismatch repair. Other reasons may lie in intratumoral genetic heterogeneity, overactivity of the so-called base excision repair mechanism, or aberrant cell cycle regulation involving mutant ataxia-telangiectasia serine/threonine kinase (ATM) or ataxia-telangiectasia and Rad3-related protein kinase (ATR), which are important proteins within the DNA damage response (DDR). These different resistance mechanisms need to be further explored, and targeting multiple signaling pathways is considered a prerequisite for successful therapy [162,163,164]. 

A particular advance in recent years has been the introduction of molecular criteria for the diagnosis and/or classification of specific tumor entities since the fourth edition of the WHO classification of central nervous system tumors in 2016 [113]. Since then, a large number of additional molecular criteria have been introduced in the current 5th edition of the WHO classification [165]. Adult and pediatric type diffuse gliomas have been classified separately due to distinct histopathological, clinical, and molecular differences. Important examples of molecular alterations in adult-type gliomas include EGFR amplification, TERT promoter mutation, and +7/-10 chromosomal copy number alterations in high-grade astrocytic tumors with IDH wild type. The presence of any of these molecular alterations is sufficient for a diagnosis of IDH wild-type glioblastoma with WHO grade 4 [166] (Table 1). In a high-grade astrocytic tumor with IDH mutation, the diagnosis of IDH-mutated astrocytoma WHO grade 4 can be made if homozygous deletions of CDKN2A/B are confirmed [167]. CDKN2A/B are regulators of CDK4/6-mediated pRB1 phosphorylation and key molecules in cell cycle control, addressing the importance of this alteration of the RB1 pathway in the diagnosis, grading, and thus prognostic evaluation of diffuse astrocytic gliomas with IDH mutation. When the above molecular alterations are present in the corresponding tumor type, they are sufficient for the diagnosis of a WHO grade 4 tumor, even in the absence of radiological or histopathological criteria of a high-grade tumor, such as necrosis or microvascular proliferation [115,166,167]. An example of a diffuse glioma of the pediatric type is the diffuse low-grade glioma with MAPK pathway alteration. Its diagnostic molecular alterations are related to the MAPK pathway, most commonly the BRAFV600E mutation. Other mutations may occur in fibroblast growth factor receptor 1 and 2 (FGFR1/2), neurotrophic receptor tyrosine kinases 1-3 (NTRK1-3), or MET protooncogene (MET) genes [168,169]. An example of the diagnostic significance of histone alterations is diffuse hemispheric glioma with the H3G34 mutation. This is a high-grade glioma of the pediatric type with WHO grade 4. Importantly, detection of an H3G34 mutation in a hemispheric glioma is sufficient for diagnosis, whether or not radiologic or histopathologic features of high-grade glioma are present [115,170] (Table 1). As an example of the diagnostic significance of DNA methylome analysis, high-grade astrocytoma with piloid features (HGAP) is a new entity within the circumscribed astrocytic glioma group. Various molecular alterations, such as mutations or gene fusions, may occur in this tumor, but so far, confirmation of the typical DNA methylation profile is the only sufficient criterion for its diagnosis [171] (Table 1). In the near future, other molecular criteria for diagnosis and prognostic assessment will be defined, and in relation to the issue of DNA methylation, hydroxymethylation is an important candidate to be further explored. Demethylation is the process of oxidative conversion of 5-methylcytosine (5mC) to 5-hydroxymethylcytosine (5hmC) by the so-called ten-eleven translocase enzymes (TETs). Reduced expression of TET enzymes and a decrease in 5hmC levels have been described in gliomas, with the reduction being much more pronounced in glioblastomas than in gliomas with a lower tumor grade [172,173]. Ascorbate, a cofactor of TET enzymes, was also significantly reduced in gliomas, with significantly lower levels in glioblastomas [173]. Further research is needed to determine whether 5-HMC has additional significance in the context of the biological evaluation of gliomas. This is also true for many other molecular pathways whose biological significance is not yet fully understood, such as alternative pathways of cell death like ferroptosis. This is an iron-dependent form of cell death in which the accumulation of reactive oxygen species (ROS) is a promoter of ferroptosis, while glutathione peroxidase 4 (GPX4) is an important inhibitor protein [174]. Although ferroptosis is generally considered an antitumor mechanism leading to tumor cell death, the role of ferroptosis in tumor biology, especially in gliomas, is still not clear. Analyses of gene risk signatures containing ferroptosis-related genes revealed a close correlation with outcome in patients with gliomas [174,175]. An important breakthrough may be the confirmation of the effect of miRNA-147a on the induction of ferroptosis with the suppression of glioma cell growth in vitro. Mechanistically, miRNA-147a inhibits Solute Carrier Family 40 Member 1 (SLC40A1), leading to iron overload and ferroptosis [176]. It is recommended to investigate the relationship between ferroptosis and other signaling pathways, such as the influence of TP53, and also to explore the possibilities of using miRNA-147a mimetics or even GPX4 inhibitors as therapeutic agents to induce ferroptosis in gliomas [174,175,176]. Another attempt at targeted induction of ferroptosis in glioma cells was made in vitro using lentiviral delivery of the circular RNA circRFN5, which has been shown to inhibit tumor cell proliferation by promoting ferroptosis via downregulation of the homeodomain transcription factor paired-related homeobox 2 (PRRX2) [177]. Together with all other observations presented in this review that show potential therapeutic relevance, circRFN5 administration is also part of a long list of efforts to establish targeted therapeutic strategies against gliomas in the future. To date, surgery, radiotherapy, and chemotherapy with alkylating agents such as temozolomide still represent the central spectrum of therapeutic modalities for patients with gliomas. Many approaches to molecularly targeted therapies have been investigated in clinical trials, and now the targeted treatment of relapsed gliomas with the BRAFV600E mutation with the BRAF kinase inhibitor dabrafenib in combination with the MEK inhibitor trametinib has provided strong evidence of clinical benefit [30,178,179]. For targeting tumors with FGFR alterations, the current evidence of clinical benefit remains to be confirmed in clinical trials [30]. For all other molecular targets, the benefit of a targeted therapy is still considered investigational and should only be tested in clinical trials [30]. In this context, it is important to keep in mind the unabated efforts to clinically test new molecular therapeutics for various targets, as shown in Table 2. These studies include those investigating new therapeutics that act against targets that have been studied multiple times in the past, such as the epidermal growth factor receptor EGFR [180]. Most of these recent studies are still at the phase I or II stage, but in several studies, there is already evidence of an objective clinical response with prolonged overall survival or even partial or complete remissions in some patients [181,182,183]. Another promising finding is that in two studies, molecular combination therapies against more than one target were well tolerated by patients, indicating a realistic perspective on using molecular combination therapies in patients with gliomas [179,182]. Therefore, all these recent studies show that continued molecular and clinical research is worthwhile to arrive at clinically applicable forms of targeted therapy [178,179,180,181,182,183,184,185,186,187] (Table 2). In summary, the review of the spectrum of molecular pathways in gliomas provides insight into two recent aspects that should be highlighted, namely, the increasing evidence of distinct pathway interconnectivity and even the finding of a prominent role of noncoding RNAs (ncRNAs) in the regulation of all molecular pathways that have a direct impact on the biological behavior of gliomas in vitro and in vivo. Many additional new insights into the molecular pathology of glioma pathways have been obtained, and we aimed to provide a comprehensive overview of all these aspects. One of the most important conclusions, widely shared in the literature, is the need to develop therapeutic strategies that target more than a single molecular fixed point to overcome resistance to therapy with single agents. Another challenge for the future will be to explore signaling pathways whose importance in glioma biology is not yet fully understood, such as alternative forms of cell death like ferroptosis. 

## Figures and Tables

**Table 1:** Importance of altered molecular pathways for the diagnosis of gliomas. Examples of diagnostic molecular criteria according to the fifth edition of the WHO classification of tumors of the central nervous system [115,165].

Tumor	Diagnostic Morphological and Molecular Criteria
Glioblastoma, IDH-wildtype, and WHO grade 4	Diffusely infiltrating astrocytic glioma with IDH-wildtype and no H3-histone alteration; at least one of the following five criteria: (1) microvascular proliferation, (2) necrosis, (3) TERT promotor mutation, (4) EGFR amplification, (5) chromosome +7/−10 copy number alterations.
Astrocytoma, IDH-mutant, and WHO grade 4	Diffusely infiltrating astrocytic glioma with IDH1- or IDH2-mutation and without 1p/19q-codeletion; at least one of the following three criteria: (1) microvascular proliferation, (2) necrosis, (3) homozygous deletion of cyclin-dependent kinase 2A or 2B (CDKN2A/B).
Diffuse low-grade glioma, and MAPK pathway altered	Diffuse glioma without histological features of a high-grade glioma and without microvascular proliferation or necrosis; genetic alterations in the MAPK-pathway; IDH-wildtype and no H3-histone alterations; no homozygous deletion of CDKN2A/B.
Diffuse hemispheric glioma and H3G34-mutant	Infiltrative glioma with hemispheric location; missense mutation of the histone H3-3A with replacement of glycine (G) by arginine or valine at position 34 of histone H3.3 (H3G34-mutation); the DNA-methylation profile can support the diagnosis.
High-grade astrocytoma with piloid features	Astrocytic glioma with a specific DNA-methylation profile of a high-grade astrocytoma with piloid features; in addition, several gene mutations may occur, such as MAPK pathway mutations or homozygous deletion of CDKN2A/B, but the diagnosis is based solely on confirmation of the specific DNA methylation profile.

**Table 2:** Important examples of current clinical trials on targeted treatment of patients with gliomas, reported in the years 2020–2023 [178,179,180,181,182,183,184,185,186,187].

First Author, Year, and Phase of Study	Therapeutic Agent	Type of the Agent
Subbiah, 2023, Phase II [178]	**Dabrafenib and Trametinib**	BRAF kinase-inhibitor and MEK-inhibitor
*The overall response rate was 54% for BRAFV600E mutant low-grade gliomas and 33% for BRAFV600E mutant high-grade gliomas. Adverse reactions occurred in the majority of patients, most commonly (>20%) fever and nausea.*		
Bouffet, 2022, Phase I/II [179]	**Dabrafenib and Trametinib**	BRAF kinase inhibitor and MEK-inhibitor
*Adequate efficacy and tolerability in BRAF V600 mutated low-grade gliomas. Significantly fewer adverse reactions were observed in patients receiving combination therapy compared to patients receiving monotherapy (22%* vs. *54%).*		
Narita, 2021, Phase I/II [180]	**Depatux-M**	EGFR-inhibitor
*EGFR-modified high-grade gliomas treated with Depatux-M in combination with TMZ; mild efficacy and tolerable safety profiles were reported.*		
Martinez-Garcia, 2022, Phase I [181]	**Crizotinib**	MET and ALK-inhibitor
*Combination of crizotinib with TMZ and radiotherapy. The combination therapy was well tolerated; median overall survival time in glioblastoma patients was 22.6 months, which was longer than expected.*		
El-Khouly, 2021, Phase I/II [182]	**Bevacizumab and** **Irinotecan and** **Erlotinib**	VEGF-inhibitor, Topoisomerase I-inhibitor, and EGFR-inhibitor
*Children with diffuse midline glioma treated with a triple combination reported to be well tolerated; median overall survival was 13.8 months with long-term survivors up to 33 months.*		
Gilbert, 2021, Phase II [183]	**Lapatinib**	Inhibitor of MGMT and ErbB1/2
*Adult patients with low- and high-grade ependymoma treated with a combination of lapatinib and TMZ. Combination therapy was well tolerated; objective response with several complete or partial remissions and reduced symptom burden was reported.*		
Lapointe, 2020, Phase I [184]	**Vistusertib**	mTOR1/2-inhibitor
*Combination of vistusertib with TMZ in GBM patients after first relapse, with favourable safety profile at doses tested.*		
Kang, 2023, Phase I [185]	**SYHA1813**	VEGF inhibitor
*In high-grade gliomas, a significant decrease in soluble VEGFR2, an increase in VEGFA, and preliminary anti-tumor activity have been observed; adverse reactions were moderate but acceptable.*		
Trippett, 2022, Phase I/II [186]	**Cobimetinib**	MEK-inhibitor
*Several tumors with MAPK changes, a clinical response was observed, especially in patients with low-grade gliomas; the therapy was well tolerated.*		
Su, 2022, Phase I/II [187]	**Vorinostat**	Histone-deacetylase- inhibitor
*Children with diffuse midline glioma were treated with vorinostat and radiation; the treatment was well tolerated but did not improve the outcome.*		

## Data Availability

No new data were created or analyzed in this study. Data sharing is not applicable to this article.

## Abbreviations

ADAM: a disintegrin and metalloproteinase; α-KG: α-ketoglutarate; AKT: protein kinase B; ALK: anaplastic lymphoma receptor tyrosine kinase; ALKBH1: AlkB-homolog 1; ALT: alternative lengthening of telomeres; Ang1: angiopoietins 1; ARL13B: ADP-ribosylation factor-like protein 13B; ATM: ataxia-telangiectasia mutated serine/threonine kinase; ATR: ataxia telangiectasia and Rad3 related protein kinase; ATRX: α-thalassemia mental retardation X-linked gene; BAK: Bcl-2 homologous antagonist; BAX: Bcl-2-associated X protein; BCL-2: B-cell lymphoma 2; BNIP3: BCL2 interacting protein 3; BRAF: rapidly accelerated fibrosarcoma type B; BRAFV600E: gene of rapidly accelerated fibrosarcoma type B with exchange of valine (V) by glutamic acid (E) at position 600; CCND2: cyclin D2; CDK4: cyclin dependent kinase 4; CDKN2A: cyclin dependent kinase 2 type A; CHC: alpha-cyano-4-hydroxycinnamate; CHD1: chromodomain helicase DNA binding protein 1; circRNA: circular RNA; c-MYC: cellular myelocytomatosis; CXCL8: chemokine C-X-C motif ligand-8; CXCR1: chemokine C-X-C motif ligand-1; DDR: DNA damage response; DMG: diffuse midline glioma; E2F: transcription factor E2F; EGFR: epidermal growth factor receptor; EGFRvIII: epidermal growth factor receptor variant III; eIF5B: eukaryotic initiation factor 5B; EP: erythropoietin; ErbB2: human epidermal growth factor receptor 2; ERK: extracellular signal regulated kinases; ESCRT: endosomal sorting complex required for transport; EVs: extracellular vesicles; EZHIP: enhancer of zeste homolog inhibitor protein; FAK: focal adhesion kinase; FAS: TNF receptor superfamily member 6; FGFR: fibroblast growth factor receptor; FMRP: fragile X mental retardation protein; FOXA2: forkhead box A2; FOXP1: forkhead box P1; G-CIMP: CpG island methylation phenotype in IDH-mutated gliomas; GLS1: glutaminase isoenzymes 1; GPCRs: G-protein coupled receptors; G-protein-RAS: G-protein-“rat sarcoma”; GPX4: glutathione peroxidase 4; H3K27ac: acetylation of histone 3 at position 27; H3K9me3: three-fold methylation of histone 3 at position 9; HATs: histone acetyltransferases; HDACs: histone deacetylases; 2-HG: 2-hydroxyglutarate; HGF/SF: hepatocyte growth factor/scatter factor; HGFR: hepatocyte growth factor receptor; HIF1: hypoxia inducible factor 1; hnRNP-A1: heterogeneous nuclear ribonucleoprotein A1; IDH1: Isocitrate dehydrogenase 1; IGF1R: Insulin-like growth factor receptor 1; JAKs: Janus kinases; KAT6A: lysine acetyltransferase 6A; KDM2B: lysine (K)-specific demethylase 2B; LATS1: large tumor suppressor 1; LncRNA: long non-coding RNA; LXR-623: liver X receptor 623; MAPK: mitogen activated protein kinase; MCTs: monocarboxylate transporters; MDM2: mouse double minute homologs 2; MEK: mitogen-activated protein extracellular regulated kinase; MET: tyrosine-protein kinase MET, syn. hepatocyte growth factor receptor (HGFR); METTL3: methyltransferase-like 3; MGMT: O6-methylguanine-DNA-methyltransferase; miRNA: microRNA; MMPs: matrix-metalloproteinases; MST1: tumor suppressors mammalian STE20-like protein kinase 1; mTOR: mammalian target of rapamycin; mTORC-1: mammalian target of rapamycin complex 1; MTSS1: metastasis suppressor protein 1; NF1: neurofibromin 1; NF2: neurofibromin 2 (merlin); ncRNA: non-coding RNA; NIX: BCL2 interacting protein 3-like; NOTCH: neurogenic locus notch homolog; NRXN3: neurexin 3; 2-OG: 2-oxoglutarate; p16^INK4^: cyclin dependent kinase inhibitor 2A-inhibits CDK4; PDGFR: platelet derived growth factor receptor; PI3K: phosphatidylinositol 3-kinase; PIP2: phosphatidyl-inositol-bisphosphate; PIP3: phosphatidyl-inositol-trisphosphate; PKM2: pyruvate kinase type 2; PLK1: polo-like-kinase 1; PRRX1/2: homeodomain transcription factor paired-related homeobox 1/2; PTEN: phosphatase and tensin homolog; PTPN14: protein tyrosine phosphatase non-receptor type 14; RB1: retinoblastoma protein 1; RhoJ: Ras homolog family member J; ROS: reactive oxygen species; RTKs: tyrosine kinases receptors; Runx1: runt-related transcription factor 1; SASH1: SAM and SH3 domain containing 1; ShcD: proto-oncogenic Src homology and collagen-protein subfamily D; SLC40A1: solute carrier family 40 member 1; SOCS3: JAK-suppressors of cytokine signaling 3; SOX4: SRY-box transcription factor 4; Src: cellular-sarcoma family kinases; STATs: signal transducers and activators of transcription proteins; TAT: transactivator of transcription; TAZ: transcriptional co-activator with PDZ-binding motif; TCF12: transcription factor 12; TEAD: transcription factors TEA domain family; TERT: telomerase reverse transcriptase; TETs: ten-eleven translocation enzymes; TGF-ß: transforming growth factor-β; TGFBI: transforming growth factor beta-induced; Tie2: tunica interna endothelial cell kinase-2; TMZ: temozolomide; TNFRs: tumor necrosis factor receptors; TP53: tumor protein 53; TRAIL: tumor necrosis factor-related apoptosis-inducing ligand; TUSC2: tumor suppressor 2-mitochondrial calcium regulator; ULK1: Unc-51-like kinase 1; UVRAG: UV irradiation resistance-associated tumor-suppressor gene; VASN: vasorin; VEGFA: vascular endothelial growth factor A; VEGFR: vascular endothelial growth factor receptor; VPS25: vacuolar protein-sorting-associated protein 25; WNT: wingless and integrin1; YAP1: yes-associated protein 1; YY1: transcription factor Yin Yang 1.
